# Artificial intelligence driven transformation of pediatric eye health education based on bibliometric analysis and a cross-sectional survey

**DOI:** 10.3389/fpubh.2026.1781008

**Published:** 2026-03-20

**Authors:** Yiman Li, Shanshan Xu, Dong Wang, Ente Wang, Jiawei Wang

**Affiliations:** Department of Pharmacy, Beijing Tongren Hospital, Capital Medical University, Beijing, China

**Keywords:** artificial intelligence, bibliometric analysis, cross-sectional survey, large language models, pediatric eye health education

## Abstract

**Background:**

Pediatric eye diseases, especially myopia and other refractive errors as well as binocular vision disorders, have evolved into a pressing global public health concern. Effective caregiver-targeted eye health education is crucial for the early prevention and long-term management of these conditions. However, conventional educational approaches often struggle to deliver actionable and accessible guidance. Emerging artificial intelligence (AI) technologies, particularly large language models (LLMs), present novel opportunities to address this gap.

**Methods:**

A mixed-methods design was conducted in this study. On one hand, a bibliometric analysis was conducted on publications related to pediatric eye health education from 2015 to 2025, using the Web of Science Core Collection and PubMed databases, with visualization implemented via Bibliometrix and CiteSpace software. On the other hand, a cross-sectional online questionnaire survey was administered to guardians of children aged 1–6 years to systematically assess their key concerns about pediatric eye diseases, knowledge levels, information sources, and preferences for educational formats.

**Results:**

A total of 111 publications were included in the bibliometric analysis, which revealed a significant surge in research output after 2020. Traditional themes such as vision screening and patient education remained central, while AI- and LLM-related topics have emerged as recent research hotspots. China and the United States led in terms of publication volume, whereas several countries with lower output demonstrated higher average citation impact. Among 328 valid survey responses, guardians showed high concern for myopia, astigmatism, and strabismus but lacked sufficient practical knowledge regarding preventive pharmacological strategies and correct eye drop administration. Additionally, child-friendly, visualized, interactive, and online educational formats were strongly preferred.

**Conclusion:**

By integrating bibliometric trends with the actual needs of caregivers, this study demonstrates that the growing role of AI and LLMs in pediatric eye health education reflects both technological advancement and unmet demands for personalized, actionable educational content. These findings provide an evidence-based foundation for the development of AI-driven pediatric eye health education tools, emphasizing the necessity of clinical oversight, ethical governance, and equitable access in future practice.

## 1 Introduction

Childhood eye diseases like myopia, amblyopia, strabismus, and congenital cataracts are a major global public health challenge, with early detection and intervention being critical to prevent irreversible vision impairment and its impact on education and quality of life. Parental awareness remains insufficient, and optimizing health education formats is crucial for improving eye health literacy and outcomes.

Multiple studies highlight significant gaps in parental knowledge about childhood eye diseases, with the majority of parents demonstrating poor or intermediate awareness, especially regarding conditions like amblyopia and congenital cataracts (1–4). Factors such as higher education, income, and having a child with an eye disease are associated with better awareness (1, 4). Preferred information sources include doctors, social media, and the internet, but traditional methods like brochures and lectures are less favored and less effective (2).

Research shows that interactive, multimedia, and digital platforms (e.g., social media, educational videos, and school-based apps) are more effective than traditional one-way communication in raising awareness, improving knowledge retention, and promoting protective behaviors (5–7). A randomized clinical trial found that weekly family health education via WeChat significantly reduced the incidence of myopia and improved parental awareness compared to standard care (7). Educational videos and digital communication tools also enhance early detection and engagement (5, 6).

Recent advances in AI and LLMs are transforming ophthalmic health education. Specialized models like EyeGPT and custom GPTs for ophthalmology can deliver accurate, understandable, and empathetic information, supporting both patients and providers (8, 9). These tools enable personalized, interactive education and can address knowledge gaps at scale, though ongoing evaluation and refinement are needed for rare diseases and complex scenarios.

This study will first employ bibliometric methods to analyze the research landscape and identify key trends in pediatric eye health education over the past decade. A cross-sectional study will then be conducted using questionnaire surveys to assess the public's actual needs regarding such communication. The results aim to provide a foundation for developing more targeted and effective pediatric eye health education strategies in the future.

## 2 Methods

### 2.1 Bibliometric analysis

#### 2.1.1 Databases and search strategy

Data for this bibliometric analysis were obtained from the Web of Science Core Collection and PubMed databases. All searches and data collection were completed on November 6, 2025, to avoid potential bias resulting from daily database updates. Literature retrieval employed a combination of Medical Subject Headings (MeSH) terms and free-text keywords, with the detailed search strategy presented in Supplementary Table 1. The search covered the period from January 1, 2015, to November 5, 2025. Only English-language articles, reviews and clinical trails were included, while other publication types such as reprints, book chapters, conference abstracts, and news reports were excluded. To ensure accuracy and reliability, two researchers independently conducted the literature search and data collection. The retrieved records were deduplicated using EndNote X9 before screening. To reduce language bias, CNKI and Wanfang were additionally searched to capture Chinese-language publications. Due to differences in publication focus, Chinese records were analyzed separately using keyword analysis.

#### 2.1.2 Data collection and analysis

Key information was extracted from the included publications, including journal name, publication year, title, authors, country or region, institutional affiliation, abstract, keywords, and references. Data visualization and analysis were performed using specialized tools, including the R-based bibliometrix package (version 4.2.3) and CiteSpace (version 6.4.R1). To ensure data quality, two authors independently carried out data extraction and analysis, thereby minimizing potential errors and bias and enhancing the credibility of the study findings.

### 2.2 Questionnaire survey

#### 2.2.1 Study participants

The study participants were guardians of children aged 1–6 years. The questionnaire survey was conducted between August 20, 2025, and November 30, 2025.

#### 2.2.2 Questionnaire design

The questionnaire was developed using the professional online survey platform Wenjuanxing. It consisted of four sections: Basic demographic information (3 items), Profiles and perceptions regarding pediatric health education (7 items), Guardian's educational needs in pediatric eye health (2 items).

The reliability and validity of the questionnaire were evaluated. The results showed that the Cronbach's α coefficient was 0.886, indicating high internal consistency. The Kaiser–Meyer–Olkin (KMO) value was 0.900, which is close to 1. Bartlett's test of sphericity yielded a chi-square value of 635.513 (Sig. = 0.000 < 0.01). These findings demonstrate that the questionnaire had good reliability and construct validity.

#### 2.2.3 Survey methods

This study adopted a cross-sectional design with convenience sampling. The required sample size was calculated using the formula: $N=\frac{Z_{\alpha /2}^{2}\cdot P\left(1-P\right)}{d^{2}}$, where *N* represents the sample size, *Z*_α/2_ is the critical value of the standard normal distribution, α is the significance level (α = 0.05), *P* is the expected proportion (*P* = 80%), and *d* is the allowable error (5%). The estimated minimum sample size was 246 participants.

The questionnaire was distributed in the form of online links and QR codes via WeChat, parenting-related groups, and public social media platforms. Eligible parents were invited to complete the questionnaire voluntarily and anonymously. Questionnaires with a response time of less than 100 seconds, patterned responses, or internal inconsistencies were excluded. The remaining questionnaires were considered valid for analysis.

#### 2.2.4 Statistical analysis

Questionnaire data were generated and exported through Wenjuanxing. A database was established using Microsoft Excel, and statistical analyses were performed using SPSS version 25.0. Categorical variables were described using frequencies, percentages, and composition ratios.

## 3 Results

### 3.1 Bibliometric analysis

#### 3.1.1 Literature search results

The initial search of the Web of Science Core Collection yielded 244 records. After screening titles and abstracts, 175 publications were excluded, resulting in 69 eligible articles. A separate search of the PubMed database identified 422 records, of which 337 were excluded following title and abstract screening, leaving 85 eligible articles. Duplicates (*n* = 43) across the two databases were then removed using EndNote software. In total, 111 publications were included in this study for the English-language databases. For the Chinese-language databases, a total of 192 records were retrieved from CNKI and 536 from Wanfang. After removing 120 duplicate records, 608 publications were included in the analysis.

#### 3.1.2 Annual analysis of publication

**Figure 1:**
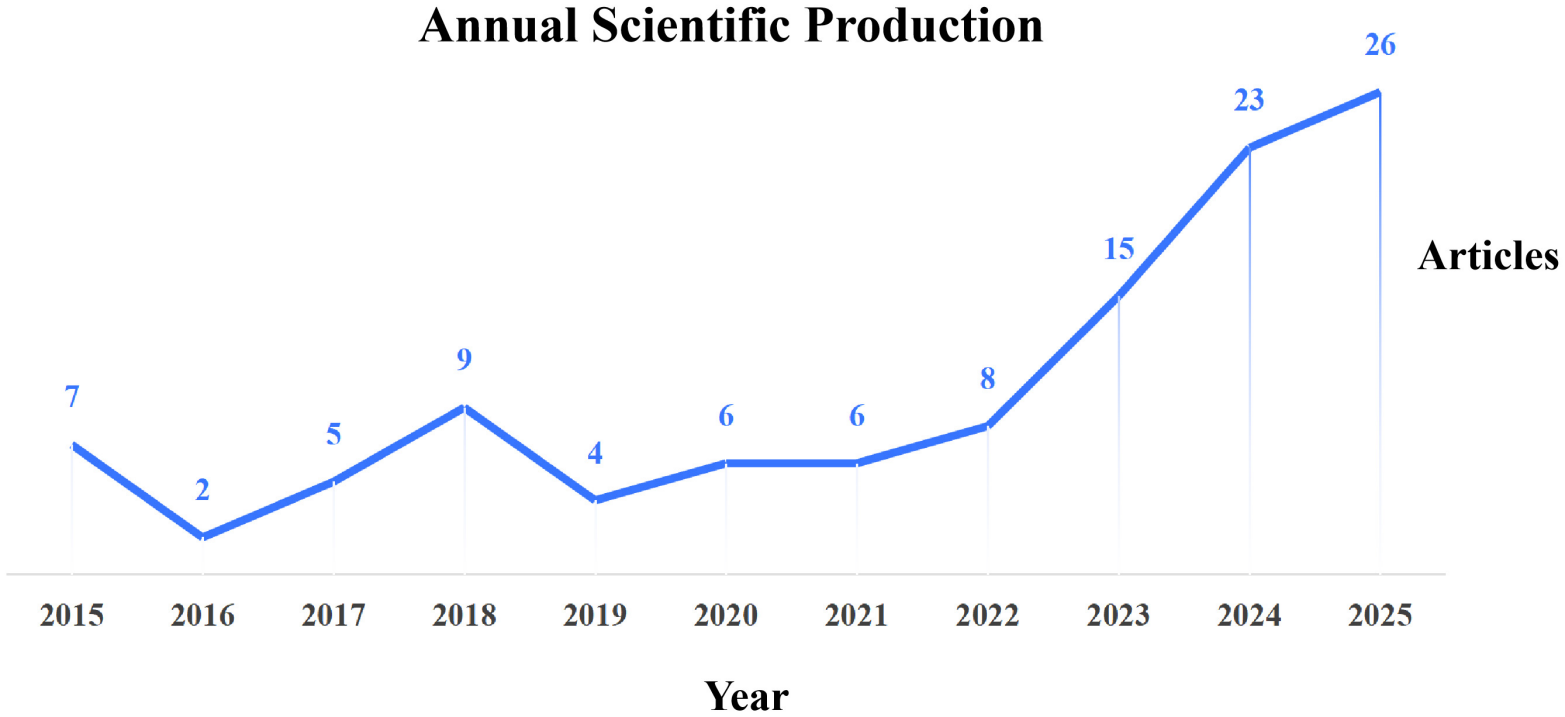
Yearly change and trend of scientific publications from 2015 to 2025.

Figure 1 shows the yearly change and trend of scientific publication from 2015 to 2025. The number of publications stayed low and changed a lot from 2015 to 2018. The publication reached a low point in 2016 with only two papers. The publication then rose slightly in 2018. The variation became smaller from 2019 to 2021. The research publication increased strongly after 2022. The number of papers grew from eight in 2022 to fifteen in 2023. The publication further rose to twenty-three in 2024 and twenty-six in 2025. Overall, this field has grown fast in recent years. Research attention has clearly increased.

#### 3.1.3 Country analysis

**Figure 2:**
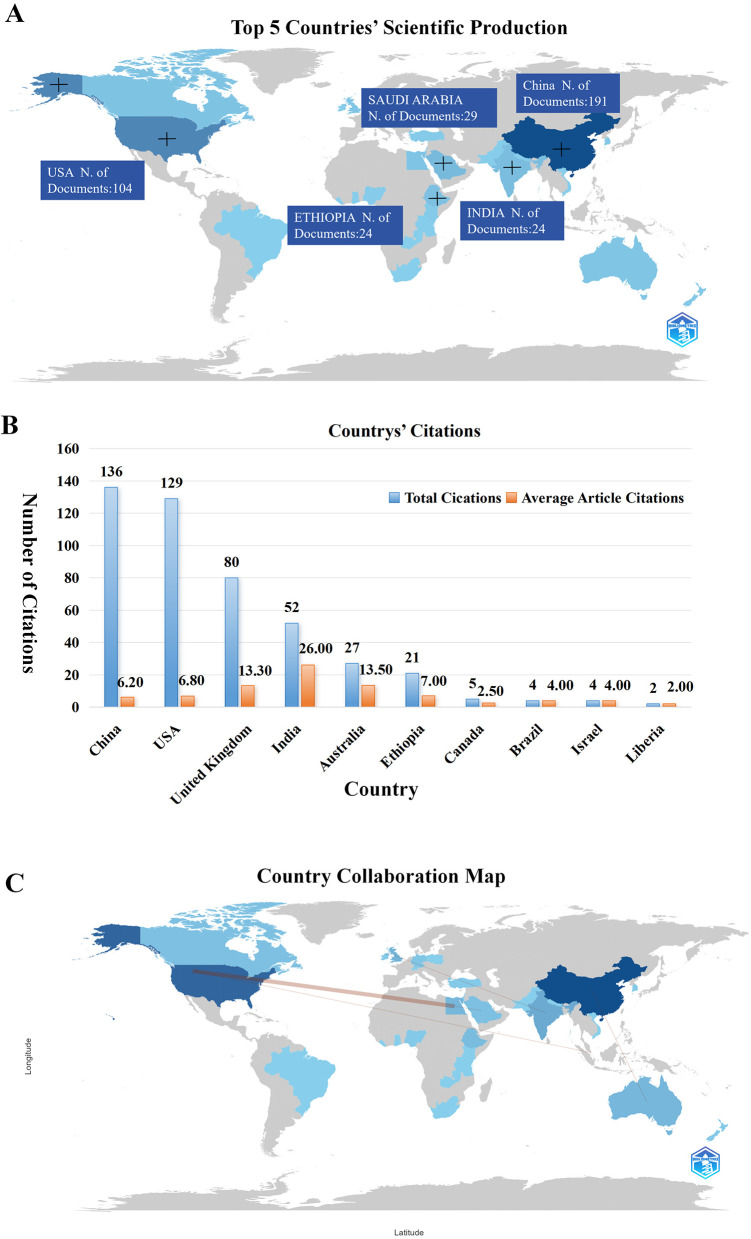
Country analysis of scientific publications. **(A)** Top 5 countries' scientific production. **(B)** Countries' citations. **(C)** Country collaboration map.

At the country level (Figure 2A), China ranked first with 191 publications, accounting for the largest share of total output, followed by the United States with 104 publications. A second group of countries, including Saudi Arabia (29 publications), India (24 publications), and Ethiopia (20 publications), showed moderate productivity, while most other countries contributed fewer than 20 papers.

In terms of citation performance (Figure 2B), China (136 citations) and the United States (129 citations) received the highest total citations, consistent with their high publication volumes. However, countries with lower output demonstrated higher average citation impact. India showed the highest average citations per article (26.0), followed by Australia (13.5) and the United Kingdom (13.3), indicating stronger influence at the individual paper level.

International collaboration analysis revealed a limited but structured network (Figure 2C). Australia maintained frequent collaborations with New Zealand and Vietnam, suggesting stable regional partnerships. Overall, the results indicate a research landscape dominated by a few high-output countries, alongside selective high-impact contributions and relatively concentrated international collaboration patterns.

#### 3.1.4 Affiliations analysis

**Figure 3:**
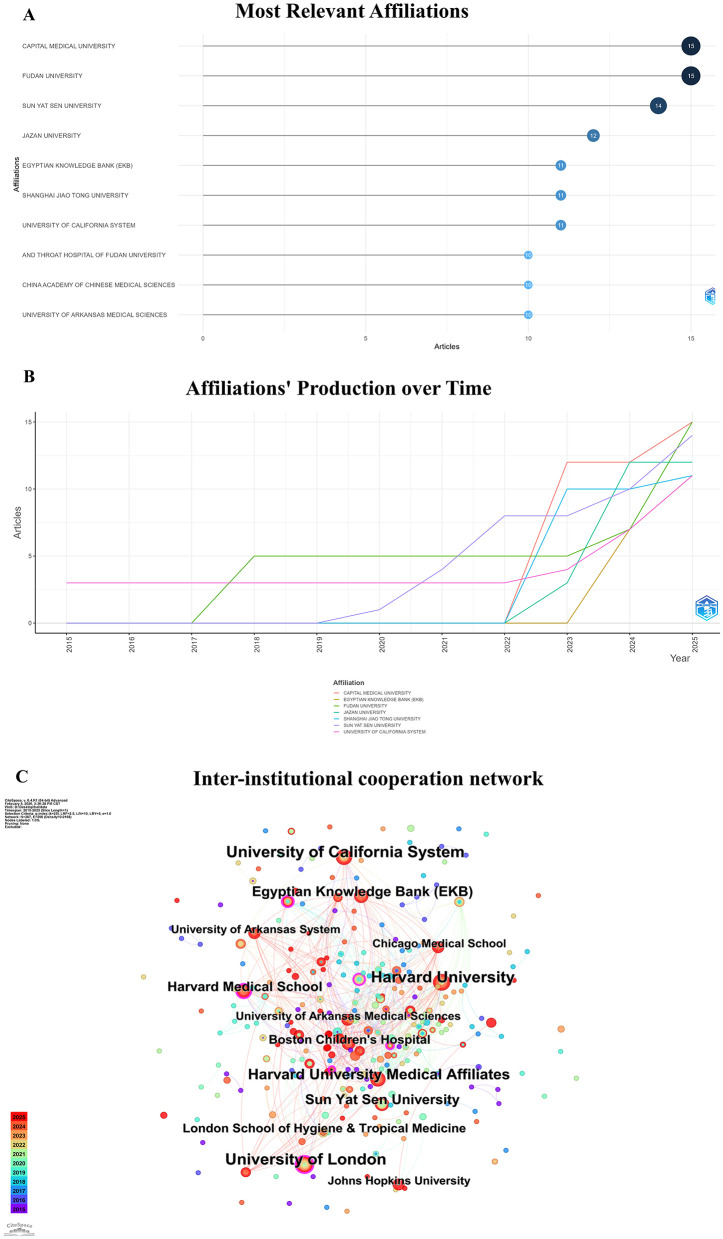
Affiliations analysis of scientific publications. **(A)** Most relevant affiliations. **(B)** Affiliations' production over Time. **(C)** Inter-institutional cooperation network.

We analyzed the academic output of relevant affiliations in this field (Figure 3). The analysis showed that research activity increased markedly after 2020. The growth rate accelerated further during the period from 2023 to 2025. In terms of the most relevant affiliations (Figure 3A), Capital Medical University and Fudan University ranked first, with 15 publications each. Sun Yat-sen University followed closely with 14 publications. Jazan University in Saudi Arabia ranked next with 12 publications. The affiliations' production over time analysis (Figure 3B) indicated that the University of California system maintained a stable level of output with recent growth. In contrast, several Chinese institutions and the Egyptian Knowledge Bank (EKB) exhibited rapid and concentrated growth over the past three years. Figure 3C reveals an international collaboration network centered around top-tier universities and medical institutions from the United States and the United Kingdom, with participation from institutions in China and Egypt. Key hubs of dense collaboration include Harvard University and Johns Hopkins University, indicating that the research activity is predominantly driven by the fields of medicine and life sciences. This pattern underscores a high-level, cross-regional characteristic of academic cooperation in this domain.

#### 3.1.5 Journal analysis

**Figure 4:**
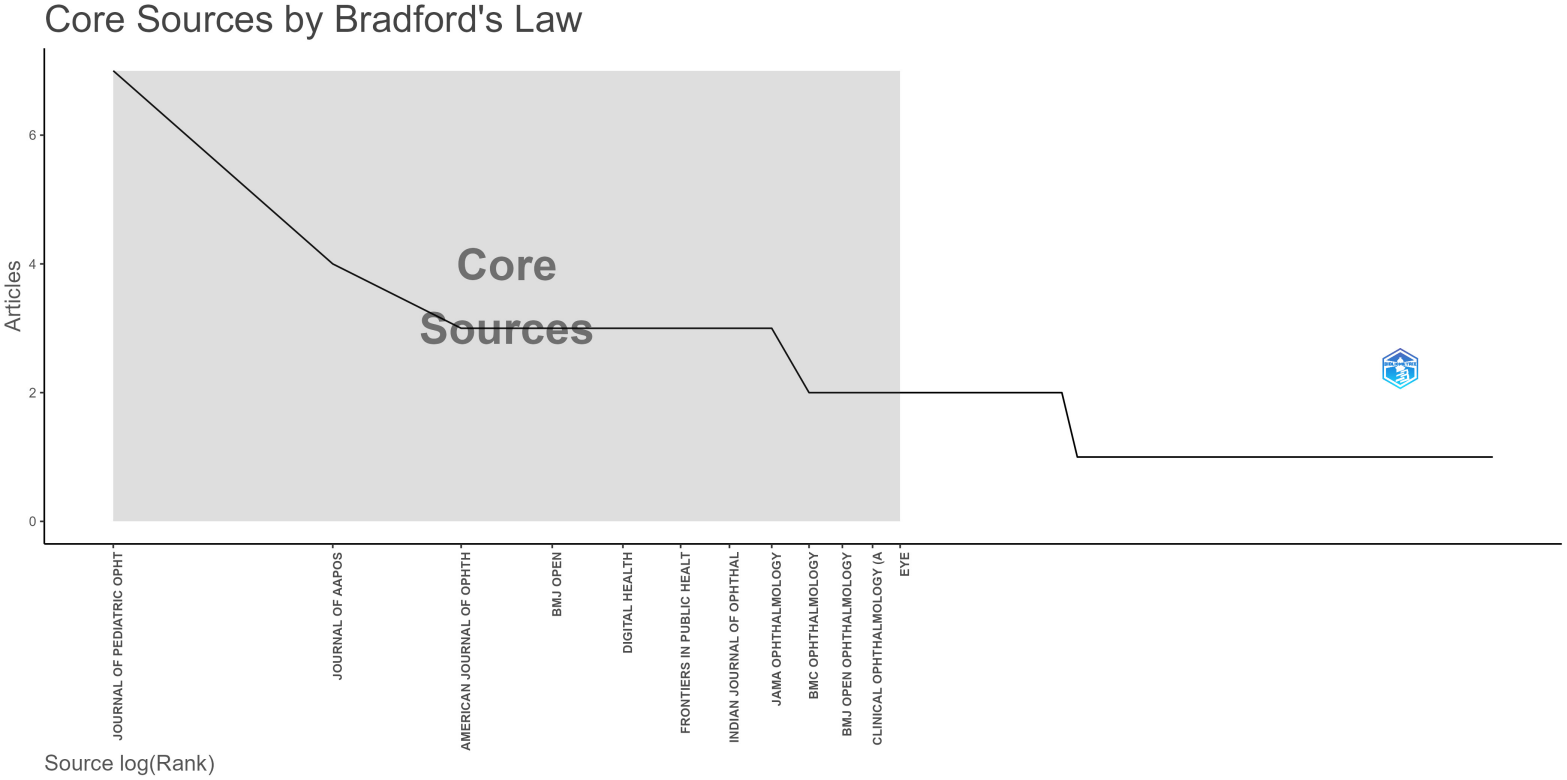
Top twelve journals that researchers consider being the primary for publication.

Bradford's Law describes the distribution of scientific articles across journals. Figure 4 showed the top twelve journals that researchers consider being the primary for publication. The largest number of publications (*n* = 7) were published in the Journal of Pediatric Ophthalmology & Strabismus. Journal of AAPOS followed with 4 articles. In the list of most cited sources, the JAMA Ophthalmology has the highest impact factor with impact factor = 9.2, followed by American Journal of Ophthalmology with an impact factor of 4.2. Furthermore, most journals are categorized as the third quartile (Q3) and the fourth quartile (Q4) in Science Citation Index Expanded (SCIE) by Journal Citation Reports (JCR) as detailed in Table 1.

**Table 1:** Most relevant journals, IF and JCR category for bibliometric insights into research trends on pediatric eye health education.

**Rank**	**Journal**	**Article (*n*)**	**IF (2024)**	**JCR category (quartile)**
1	Journal of Pediatric Ophthalmology & Strabismus	7	0.9	Ophthalmology: SCIE (Q4) Pediatrics: SCIE (Q4)
2	Journal of AAPOS	4	1.3	Ophthalmology: SCIE (Q4) Pediatrics: SCIE (Q4)
3	American Journal of Ophthalmology	3	4.2	Ophthalmology: SCIE (Q1)
4	BMJ Open	3	2.3	Medicine, General & Internal: SCIE (Q4)
5	Digital Health	3	3.3	Health Care Sciences & Services: SCIE (Q3) Health Policy & Services: SCIE (Q3) Public, Environmental & Occupational Health: SCIE (Q3) Medical Informatics: SCIE (Q4)
6	Frontiers In Public Health	3	3.4	Public, Environmental & Occupational Health: SCIE (Q3)
7	Indian Journal of Ophthalmology	3	1.9	Ophthalmology: SCIE (Q4)
8	JAMA Ophthalmology	3	9.2	Ophthalmology: SCIE (Q1)
9	BMC Ophthalmology	2	1.7	Ophthalmology: SCIE (Q3)
10	BMJ Open Ophthalmology	2	2.2	Ophthalmology: SCIE (Q4)

IF, impact factor; JCR, Journal Citation Reports; SCIE, Science Citation Index Expanded.

#### 3.1.6 Authors analysis

**Figure 5:**
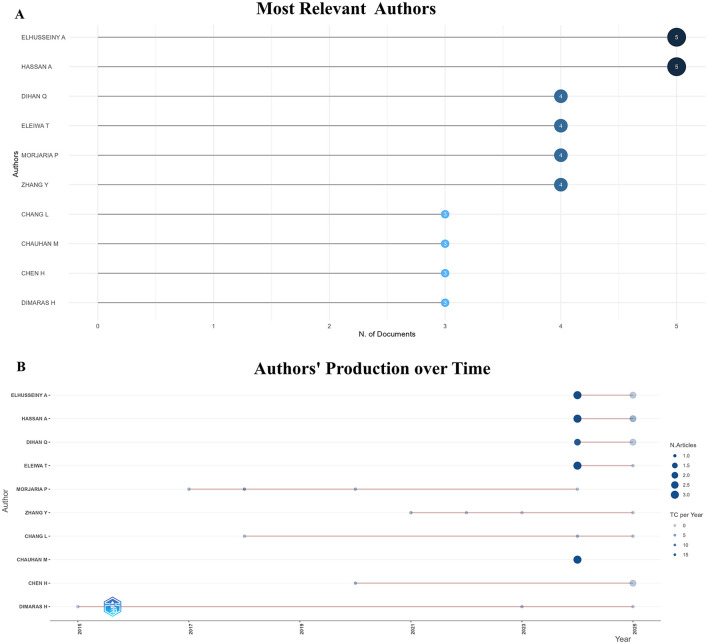
Authors analysis. **(A)** Most relevant authors. **(B)** Authors' production over time.

Among the top ten authors in the field of pediatric Eye Health Education, Elhusseiny A and Hassan A stand out with 5 articles, respectively (Figure 5A), while other researchers such as Dihan Q and Eleiwa T form a stable core author group. Furthermore, the authors' production over time in Figure 5B indicates that overall publication activity in this field has increased noticeably since 2020, with the output of major authors remaining active during this period. Dimaras H started their publication activity in 2015 and remaining active until now, indicative of sustained engagement and enduring interest. In 2024, Elhusseiny A, Hassan A, Eleiwa T and Chauhan M each published 3 articles in this field and the total citations (TC) per year of each author was 18.

#### 3.1.7 Keywords analysis

##### 3.1.7.1 Keywords co-occurrence analysis

**Figure 6:**
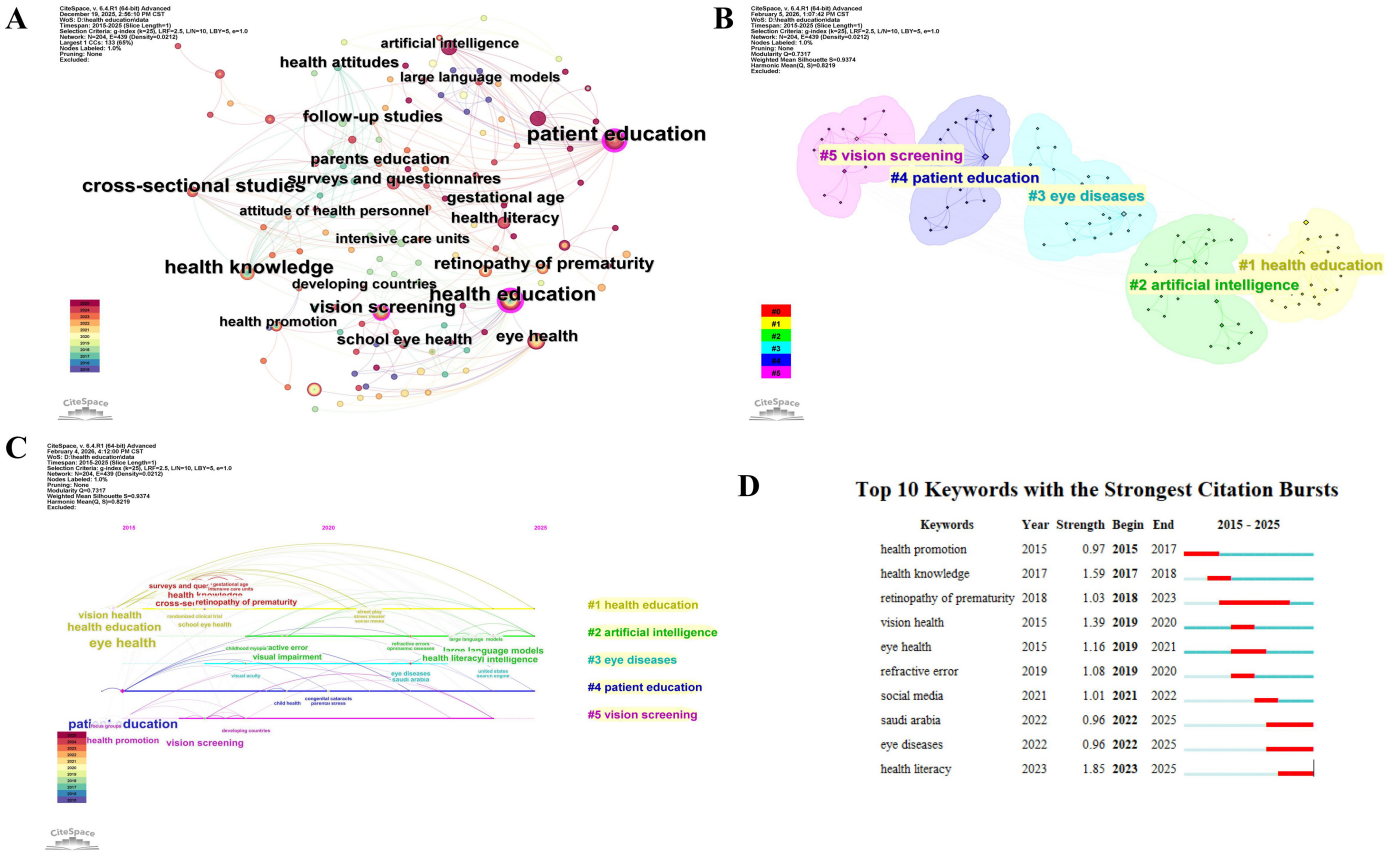
Keywords analysis. **(A)** Keyword co-occurrenc visualization analysis on pediatric eye health education. **(B)** Keyword cluster analysis on pediatric eye health education. **(C)** The timeline view of keywords analysis on pediatric eye health education. **(D)** The top 10 Keywords with the strongest citation bursts.

High-frequency keywords describe a hot issue in a research area, while high-centrality keywords show the status and importance of the relevant study material in the research field. Table 2 showed the top 10 keywords in pediatric eye health education. As shown in Table 2, the most frequently occurring keyword is eye health (frequency: 22), and the one with the highest centrality is patient education (centrality: 0.31). Figure 6A showed the network of co-occurring keywords, which obtained 457 nodes, 784 links, and a network density of 0.0075.

**Table 2:** Top 10 keywords in frequency and centrality on pediatric eye health education.

**Rank**	**Frequency**	**Year**	**Keywords**
1	22	2015	Eye health
2	19	2015	Patient education
3	13	2015	Health education
4	9	2015	Vision health
5	8	2017	Vision screening
6	7	2024	Artificial intelligence
7	7	2024	Large language models
8	6	2017	Cross-sectional studies
9	6	2017	Health knowledge
10	6	2023	Health literacy
**Rank**	**Centrality**	**Year**	**Keywords**
1	0.31	2015	Patient education
2	0.26	2015	Health education
3	0.12	2017	Vision screening
4	0.10	2017	Health knowledge
5	0.08	2015	Eye health
6	0.08	2018	Retinopathy of prematurity
7	0.07	2023	Health literacy
8	0.06	2024	Artificial intelligence
9	0.05	2017	Cross-sectional studies
10	0.05	2022	Eye diseases

##### 3.1.7.2 Keywords clusters analysis

We clustered the co-occurring keywords to find hot research topics, and the results are shown in Figure 6B. The network map has a substantial clustering structure, and the structure is persuasive, according to the Q value produced by clustering keywords in this study, which is 0.7317 (>0.3), and the S value, which is 0.9374 (>0.7). The top 6 cluster label groups are #1 health education, #2 artificial intelligence, #3 eye diseases, #4 patient education, #5vision screening.

##### 3.1.7.3 Keywords timeline analysis

The horizontal line represents the year in which the paper was published, and the vertical line shows different clusters. Each node represents keywords, and the larger the node, the higher the frequency of their occurrence. As shown in Figure 6C, cluster health education, patient education and vision screening have the longest research period. It is noteworthy that large language models and artificial intelligence under the artificial intelligence cluster have emerged as research hotspots in recent years.

##### 3.1.7.4 Keywords citation burst analysis

Keyword burst detection helps to identify emerging research trends within a specific timeframe. Based on a co-occurrence network analysis, a citation burst analysis of keywords was performed, from which the top 10 keywords with the strongest burst strength were identified (Figure 6D). In the resulting visualization, “Begin” and “End” denote the start and conclusion years of the burst period. “Strength” indicates the burst intensity, reflecting the keyword's temporal prominence and sustained attention within the dataset. Keywords exhibiting higher burst strength typically signal rapidly evolving focal points during their active periods. Analyzing these prominent keywords thus effectively reveals the prevailing and dynamic research hotspots within the field.

##### 3.1.7.5 Thematic map

**Figure 7:**
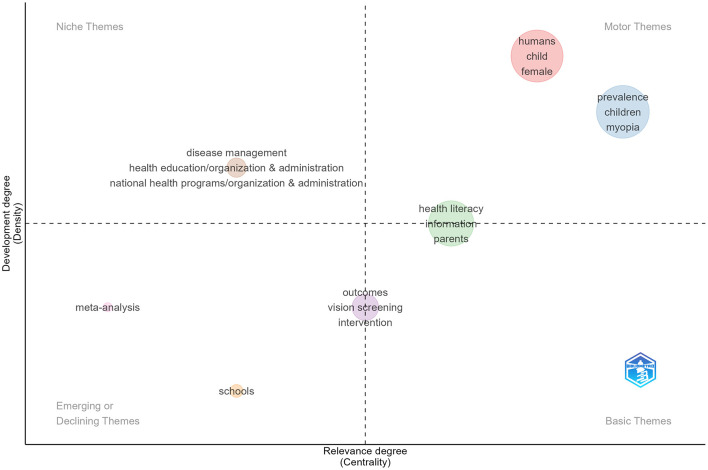
Thematic Map. Themes are sorted into four types. Words in the upper right corner (first quadrant) are motor themes, in the upper left corner (second quadrant) are niche themes, in the lower left (third quadrant) are emerging or declining themes, in lower right (fourth quadrant) are basic themes.

Using four complementary science mapping tools, we visualized the knowledge structure and research frontiers in pediatric eye health education (Figure 7). The strategic diagram organizes themes into four quadrants. The circles symbolize the themes, with their size reflecting the volume or significance of the research and their positioning indicating the stage of development and the level of relevance. The upper-right quadrant contains motor themes (myopia prevalence, children, myopia), representing central, well-developed research. The upper-left quadrant holds niche themes (disease management, health education, national programs), which are internally developed but less connected. The lower-left quadrant includes emerging/declining themes (health literacy, information, parental involvement), showing limited development. The lower-right quadrant consists of basic themes (meta-analysis, outcomes, vision screening, intervention, school programs), with strong external linkages but needing internal elaboration.

### 3.2 Questionnaire survey

#### 3.2.1 Basic demographic information

A total of 336 questionnaires were collected. According to the predefined exclusion criteria, 18 questionnaires were deemed invalid. Ultimately, 328 valid questionnaires were included in the analysis, yielding an effective response rate of 97.62%. According to the geographical data of the 328 valid responses from the Wenjuanxing platform, Beijing residents accounted for 28.66% of the total, while 71.34% were from non-Beijing areas, covering 27 provinces across China (detailed geographical distribution of the sample in the revised manuscript (Supplementary Table 2). The basic characteristics of the participants are presented in Table 3. Children aged 1 year constituted the largest group (32.32%), followed by those aged 6 years (23.48%). Girls accounted for 52.13% of the sample, while boys accounted for 47.87%, indicating a balanced sex distribution. Most questionnaires were completed by mothers (70.12%), followed by fathers (22.87%). Grandparents and other guardians together accounted for >7% of respondents, suggesting that mothers were the primary caregivers participating in the survey.

**Table 3:** Basic characteristics of the participants.

**Profiles**	**Frequency**	**Proportion**
Age of the children		
1	106	32.32%
2	43	13.11%
3	47	14.33%
4	24	7.32%
5	31	9.45%
6	77	23.48%
Sex of the children		
Male	157	47.87%
Female	171	52.13%
Type of the guardian		
Mother	230	70.12%
Father	75	22.87%
Grandmother	5	1.52%
Grandfather	6	1.83%
Others	12	3.66%

#### 3.2.2 Profiles and perceptions regarding pediatric health education

As shown in Table 4, guardians demonstrated moderate engagement with pediatric health education. More than half reported occasional exposure when encountering relevant content (53.66%), while one third engaged frequently and proactively (33.23%). Online platforms were the predominant information source (83.23%), followed by medical institutions (58.23%), indicating a strong dependence on digital media supplemented by professional guidance. Limited daily exposure (53.35%) and lack of time (35.67%) were the main reasons for insufficient knowledge, whereas lack of interest was uncommon.

**Table 4:** Profiles and perceptions regarding pediatric health education.

**Profiles**	**Frequency**	**Proportion**
1.Attention to pediatric health education		
Frequently and proactively view	109	33.23%
Occasionally view when encountered	176	53.66%
Only view when the child is ill	36	10.98%
Rarely view	7	2.13%
2.Information sources		
Medical institutions (physicians/pharmacists; brochures/videos)	191	58.23%
Online platforms (e.g., Baidu, Douyin/TikTok, WeChat, Xiaohongshu/Little Red Book)	273	83.23%
Professional books	98	29.88%
Experiences shared by relatives or friends	137	41.77%
3.Reasons for limited knowledge		
Lack of time	117	35.67%
Lack of interest	30	9.15%
Limited daily exposure	175	53.35%
Perceived as not useful	40	12.20%
Already knowledgeable	71	21.65%
4.Frequency of pediatric health education		
Frequently	73	22.26%
Occasionally	183	55.79%
Rarely or never	72	21.95%
5.Perceived necessity		
Highly necessary	252	76.83%
Moderately necessary	59	17.99%
Not necessary	15	4.57%
Unclear	2	0.61%
6.Preferred formats		
Children's picture books	253	77.13%
Animated videos	256	78.05%
Expert lectures	81	24.70%
Interactive games	210	64.02%
7.Areas for improvement		
Lack of engagement	148	45.12%
Not age-appropriate	155	47.26%
Overly complex explanations	198	60.37%
Low attractiveness of content	175	53.35%
Concerns about accuracy	114	34.76%

Most guardians provided pediatric health education to their children only occasionally (55.79%). Despite this, perceived necessity was high, with 76.83% considering such education highly necessary. Children's picture books (77.13%) and animated videos (78.05%) were the most preferred formats, followed by interactive games (64.02%). Key areas requiring improvement included overly complex explanations (60.37%), insufficient attractiveness (53.35%), and limited age appropriateness (47.26%). Overall, these findings underscore the need for accessible, engaging, and developmentally appropriate pediatric health education materials delivered through commonly used digital platforms.

#### 3.2.3 Guardian's educational needs in pediatric eye health

**Figure 8:**
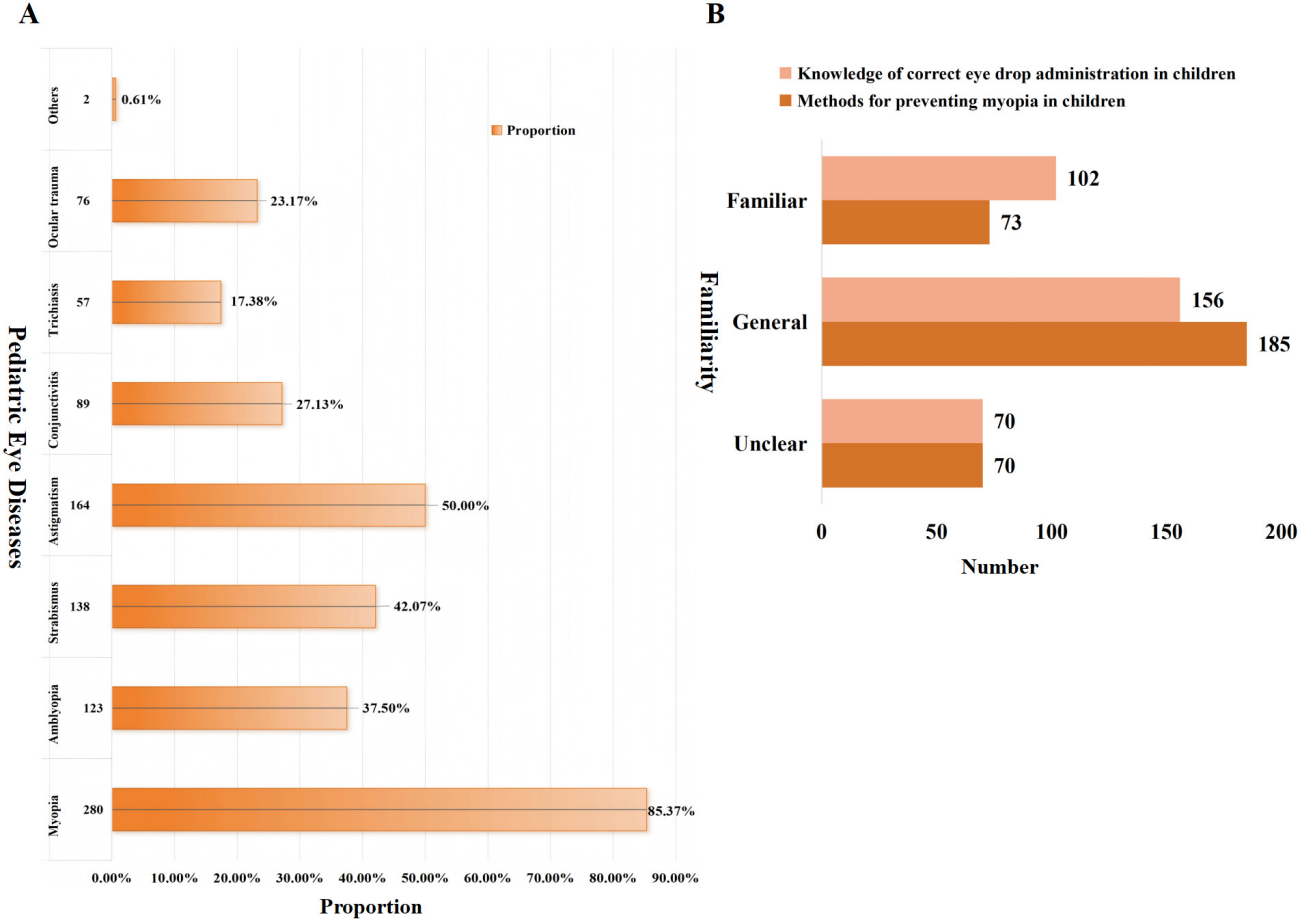
Guardian's educational needs in pediatric eye health. **(A)** Primary ocular health concerns in children. **(B)** Level of familiarity in pediatric eye topics.

Figure 8 presents guardian's concerns regarding pediatric eye conditions and their self-reported knowledge of eye care. As shown in Figure 8A, Myopia was the most commonly reported concern (280, 85.37%), followed by astigmatism (164, 50.00%), strabismus (138, 42.07%), and amblyopia (123, 37.50%). Lower levels of concern were reported for conjunctivitis (89, 27.13%), ocular trauma (76, 23.17%), and trichiasis (57, 17.38%), while other conditions were rarely selected (2, 0.61%). In respect of knowledge (Figure 8B), 56.40% of guardians reported a general understanding of preventive medications or methods for childhood myopia, whereas 21.34% reported limited understanding. For correct eye drop administration in children, 47.56% reported general understanding and 31.10% reported being very familiar.

#### 3.2.4 Guardian's education level and health education preference profile

As shown in Table 5, analysis of guardian preferences reveals a strong consensus across all education levels regarding optimal methods for delivering medical knowledge to children. “Children's picture books” and “animated videos” were the most favored channels, with preference for these visually engaging formats increasing with guardian education level (peaking at 91.11% and 82.22% for picture books and videos among postgraduates, respectively). “Interactive educational games” also received consistently high approval (all groups >60%), while “expert-led talks” were the least preferred method (< 30% across all groups).

**Table 5:** Guardian's education level and health education preference profile.

**Guardian's education level**	**High school or below**	**Associate degree**	**Bachelor's degree**	**Master's degree or above**
Most appropriate methods for delivering medical knowledge to children				
Children's picture books	13 (61.90%)	32 (74.42%)	126 (72.41%)	82 (91.11%)
Animated videos	12 (57.14%)	33 (76.74%)	137 (78.74%)	74 (82.22%)
Expert-led talks/lectures	6 (28.57%)	13 (30.23%)	50 (28.74%)	12 (13.33%)
Interactive educational games	13 (61.90%)	27 (62.79%)	107 (61.49%)	63 (70%)
Other (Please Specify)	1 (4.76%)	0 (0.00%)	1 (0.57%)	1 (1.11%)
Key areas for improvement in child-friendly medical science content				
Presentation is too dull, failing to engage children's interest	9 (42.86%)	19 (44.19%)	69 (39.66%)	51 (56.67%)
Content is not age-appropriate for my child (either too simple or too advanced)	7 (33.33%)	22 (51.16%)	75 (43.10%)	51 (56.67%)
Explanations are overly complex and difficult for children to comprehend	8 (38.10%)	28 (65.12%)	99 (56.90%)	63 (70%)
Content is not sufficiently engaging; children lack motivation to view/read it voluntarily	9 (42.86%)	26 (60.47%)	86 (49.43%)	54 (60%)
Concerns about content accuracy and potential misinformation	4 (19.05%)	13 (30.23%)	57 (32.76%)	40 (44.44%)
Other (please specify)	2 (9.52%)	0 (0.00%)	1 (0.57%)	1 (1.11%)

Regarding areas for improvement, “overly complex explanations” were identified as the primary shortcoming, a concern that heightened with guardian education (70.00% among postgraduates). Other significant issues included “presentations failing to engage children's interest” and “content lacking age-appropriateness”. Notably, guardians with postgraduate degrees expressed the highest concern over “content accuracy and potential misinformation (44.44%)”.

## 4 Discussion

This study combines bibliometric analysis with a cross-sectional survey of guardians. The results indicate a pivotal shift in pediatric eye health education. The field is moving away from traditional, static information delivery. It is transitioning toward adaptive, technology-enabled models. A clear discrepancy exists among parents and guardians. They express high concern regarding refractive and binocular vision disorders. However, they demonstrate limited operational knowledge about medication use and behavioral management. This pattern aligns closely with findings from multinational Knowledge, Attitude, and Practice (KAP) studies. These studies consistently report a common gap: caregivers are often aware of a condition, yet they lack the practical know-how for effective management (10–12). Existing KAP studies across regions consistently show that caregivers hold positive attitudes toward children's eye health but often have insufficient or uneven knowledge, which may limit effective preventive practices. Similar patterns were observed in our survey, where strong interest in eye health education coexisted with heterogeneous knowledge levels. Unlike traditional KAP studies focusing mainly on awareness and behaviors, our study emphasizes caregivers' preferences for educational formats and digital tools, suggesting that technology-based education may help translate positive attitudes into more effective practices across different settings (13–15).

From a bibliometric perspective, this study identified a significant increase in relevant publications after 2020. Pediatric eye health has become a consistently growing research focus within the field, reflecting growing global attention to pediatric eye health education in parallel with the rising prevalence of childhood myopia and increasing recognition of early-life visual health as a public health priority (16–18). Within this research domain, scholarly output is concentrated in a limited number of countries. China and the United States are primary examples. However, some nations with lower publication volumes demonstrate relatively high citation impact. This indicates their contributions are focused and influential.

The keyword co-occurrence and clustering analysis in this study reveals that traditional themes, including vision screening, patient education, and school-based interventions, remain foundational. However, topics related to AI and LLMs are emerging rapidly. This finding aligns with bibliometric results from other ophthalmic fields like myopia, diabetic retinopathy and pediatric eye diseases (17–19). Across the broader medical education literature, publications on AI and LLMs have shown a sharp rise in the past two years. Contributions from countries like China and the United States are particularly prominent. Tools such as ChatGPT are now regarded as a significant new frontier in both medical education and digital health (20–24). This indicates that AI-driven education is transitioning from peripheral exploration to systematic integration, emerging as a key trend in pediatric eye health research.

This study conducted a visual analysis of the Chinese-database (CNKI and Wanfang) in the field of children's eye health (Supplementary Figure 1). The keyword co-occurrence network (*N* = 201, *E* = 490) is well-structured, with high clustering quality (*Q* = 0.5936, *S* = 0.8849), forming core themes such as amblyopia, regression analysis, students, and health promotion. Temporal evolution analysis indicates that the research focus has shifted from early-stage amblyopia care and intervention, through mid-term comprehensive vision assessment, to a current predominant emphasis on “myopia prevention and control” and “health promotion.” Burst detection further confirms that “myopia prevention and control” and “health promotion” are the strongest current research frontiers, highlighting a field-wide transition from disease management to comprehensive public health prevention strategies. Notably, differences were observed between Chinese-language and English-language literature trends. Although Chinese authors contribute substantially to international publications indexed in Web of Science and PubMed, research published in domestic databases such as CNKI and Wanfang places less emphasis on artificial intelligence or large language models. Instead, these studies predominantly focus on clinical practice, public health interventions, and vision health education strategies tailored to local healthcare systems. This discrepancy does not indicate a lack of technological engagement but rather reflects a dual-track dissemination pattern. Chinese researchers often present technologically advanced or methodologically innovative studies in international journals, while practice-oriented and policy-relevant research is more frequently published in domestic journals. Such contextualized publication behavior has been reported in other bibliometric studies from non-English-speaking regions and highlights the importance of incorporating multilingual databases when interpreting global research trends.

The questionnaire findings offer essential real-world context for interpreting these trends. Guardians generally express high concern about refractive and binocular vision abnormalities, particularly myopia, astigmatism, and strabismus. However, their knowledge remains limited in actionable areas, such as medication use, eye drop administration, and long-term behavioral management. This aligns with the “aware of the problem—unclear about the cause—unfamiliar with solutions” pattern identified in parental studies on pediatric eye health in regions like Ethiopia and Saudi Arabia (10, 25). In the present study, guardians show strong demand for pediatric eye health education, prefer formats that are “child-friendly, visualized, and highly interactive,” and primarily rely on online platforms for information. This is consistent with existing literature, where parents mainly obtain pediatric eye health information through physicians and online/social media (26, 27). Notably, previous systematic reviews on pediatric ophthalmic patient education interventions indicate that even one-way educational formats—such as traditional printed materials, computer programs, or animated videos—can moderately improve parental knowledge, medication adherence, anxiety levels, and certain visual outcomes (28–30). However, most of these interventions are one-time and static, lacking the ability to adjust in real time based on the audience's understanding level and specific contexts. Furthermore, multiple studies have highlighted that existing online patient education materials are overly complex and use obscure language (31, 32). This suggests that relying solely on traditional educational models is insufficient to meet parents' practical needs for “actionable” and “comprehensible” information. Against this backdrop, AI and LLM are not merely technical possibilities. They represent a necessary evolution driven by clear user needs. In the fields of medicine and health education, LLM has been widely discussed for applications such as personalized learning plans, real-time feedback, intelligent Q&A, and scenario simulation. These tools can deliver continuous interaction, on-demand explanations, and clinical scenario-driven learning experiences (24, 33, 34).

The integration of AI and LLMs offers transformative potential for pediatric eye health education by facilitating highly specific and interactive scenarios. In terms of practical application, AI enables personalized interactive Q&A sessions; for instance, by employing Retrieval-Augmented Generation (RAG), LLMs can simplify complex concepts like “axial elongation” into child-friendly metaphors, such as “the eye growing like a stretching balloon.” Furthermore, immersive simulation learning—integrating AI with Virtual Reality (VR)—allows children to experience simulated visual impairments (e.g., myopic blur), thereby fostering a deeper understanding of eye protection. From a technical perspective, a robust implementation plan involves a three-tier architecture: a knowledge layer grounded in authoritative clinical guidelines, a processing layer utilizing fine-tuned specialized models like EyeGPT, and a user interface featuring digital avatars for multimodal guidance (8, 35). Case studies underscore the feasibility of this approach; recent research indicates that optimized LLMs significantly outperform traditional medical brochures in the readability of myopia-related content, while AI-driven screening initiatives at institutions like the Ninth People's Hospital have already validated the clinical efficacy of intelligent vision assessments in pediatric populations (36).

It is important to emphasize that the rapid expansion of AI and LLM in health and medical education brings significant ethical and practical challenges (20, 23). Potential risks of intelligent tools in data security, content accuracy, and system fairness must be addressed. Relevant tools should be developed and supervised collaboratively by ophthalmic and pharmaceutical professionals. Special attention should be paid to the accessibility of resource-constrained groups. This avoids exacerbating existing health inequalities due to technological application (37, 38). In the context of this study, the deployment of AI and LLMs depends not only on technical performance but also on robust ethical and feasibility safeguards. Firstly, pediatric ophthalmic data (including high resolution images, refractive measurements, and records related to learning and visual behaviors) are highly sensitive and may pose substantial privacy and security risks during collection, storage, cross-institutional sharing, and model training (39, 40). It is therefore essential to adopt a privacy-by-design approach from the outset, incorporating rigorous de-identification, access control, and encryption, and ensuring compliance with applicable regulations on medical data and children's data protection (41). Secondly, algorithmic fairness is critical for building trustworthy and widely adoptable AI systems. Prior work has shown that medical AI models trained on unbalanced datasets can exhibit substantial performance disparities across patient groups or regions, thereby unintentionally exacerbating health inequities (42, 43). In pediatric eye health and public health applications, if models are primarily developed using data from urban or high-resource settings, their predictions and recommendations for children in rural, underserved, or minority populations may be less accurate (44, 45). Accordingly, model development and evaluation should incorporate data from diverse regions and demographic groups, report stratified performance metrics, and explore debiasing techniques and fairness constraints to mitigate potential discriminatory impacts (42, 46). Thirdly, in resource-constrained or connectivity-limited settings (e.g., primary schools or remote screening sites), AI/LLM tools that rely solely on cloud computing and stable internet connections may be difficult to implement in practice (47). On the one hand, lightweight and parameter-efficient adaptation techniques can be used to deploy smaller, task-specific models on local or edge devices, thereby reducing hardware and bandwidth requirements (48). On the other hand, privacy-preserving collaborative frameworks such as federated learning can enable multiple institutions to jointly update models without sharing raw pediatric eye data, balancing privacy protection, model performance, and heterogeneous resource levels. In addition, designing simplified user interfaces and local caching strategies for low-bandwidth or even offline environments can improve the accessibility and usability of LLM-based tools in real-world public health settings (44).

In the context of child eye-health education, the real-world adoption of AI-based educational tools depends not only on algorithmic performance but also on users' trust and acceptance (49). Prior research on educational technology and intelligent tutoring systems shows that parents' and teachers' perceived usefulness and ease of use, as well as their perceived risks, are key predictors of their intention to adopt and continue using such tools (50). Therefore, when promoting LLM-based interventions for children's visual health, it is important to enhance user trust through transparent model explanations, controllable human-AI interaction, and clear communication of errors and uncertainties. Regarding implementation pathways, LLMs need to be adapted to children's cognitive developmental stages by tailoring the user interface and language output. For example, simplifying instruction structures, controlling information density, using concrete examples and visual cues, and providing step-wise feedback and error-correction support (51, 52). Evidence from child-computer interaction and intelligent tutoring systems suggests that aligning content difficulty and linguistic complexity with children's cognitive load improves learning outcomes and reduces frustration. Overall, only by adequately addressing data privacy, algorithmic fairness, and resource heterogeneity can AI and LLM tools enhance pediatric eye health and optimize resource allocation without introducing new ethical risks or reinforcing existing inequities.

The novelty of this study lies in three main aspects. First, it combines bibliometric analysis with a cross-sectional survey, allowing for both a systematic mapping of global research trends in pediatric eye health education and an in-depth understanding of caregivers' preferences and information needs in China. Second, this study incorporates an artificial intelligence perspective, exploring the potential application of AI tools in pediatric eye health education, an area rarely addressed in previous studies. Third, the survey covered caregivers across multiple provinces and varying educational backgrounds, enhancing the conceptual and methodological relevance of the findings. Unlike existing mixed-methods research, which often focuses primarily on evaluating specific interventions or educational programs, our study emphasizes understanding user needs and provides a transferable methodological framework to guide future cross-cultural and cross-regional research in pediatric eye health education.

This study has several limitations. Firstly, the bibliometric analysis relied on data extracted from Web of Science and Pubmed, while comprehensive, these databases may not index all relevant publications, particularly those in regional journals or in languages other than English, potentially introducing selection bias. Secondly, the search strategy, though carefully designed, depends on the selection of keywords and Boolean operators. Some pertinent studies might have been omitted if they used unconventional terminology. Thirdly, the questionnaire survey employed convenience sampling and was conducted primarily online, which may limit generalizability to populations with lower digital access or literacy. Fourthly, knowledge levels were self-reported and may not fully reflect actual understanding or behavior. Finally, the sample of this study was predominantly composed of mothers, with a limited proportion of fathers and grandparents. While this reflects the central role of mothers in pediatric healthcare and disease management within the Chinese context, it also constrains the depth of comparative analysis regarding cognitive differences among various caregiving roles (53). At the same time, grandparental involvement in daily upbringing is surging in China, making it a critical and routine caregiving model, especially with the high engagement of maternal grandmothers (54). It is essential that future AI-driven child health tools accommodate various caregivers by incorporating “senior-friendly” designs, such as voice-activated features and simplified interfaces. By adopting a holistic family-based education strategy, these tools can bridge the digital gap between generations and lead to more effective improvements in children's health practices (55).

In conclusion, this research illustrates that pediatric eye health education constitutes a growing research domain, marked by rising technological integration alongside ongoing deficiencies in caregiver health literacy. By framing AI and LLMs as a core innovation underpinned by both bibliometric patterns and caregiver-identified needs, the results deliver a solid, evidence-based basis for designing more impactful, tailored, and scalable strategies for pediatric eye health education.

## Data Availability

The original contributions presented in the study are included in the article/Supplementary material, further inquiries can be directed to the corresponding author.
