# Supplementary material for: Artificial intelligence driven transformation of pediatric eye health education based on bibliometric analysis and a cross-sectional survey
Source: Front Public Health. 2026 Mar 20;14:1781008. doi: 10.3389/fpubh.2026.1781008 (PMC13047839; doi:10.3389/fpubh.2026.1781008)
Supplement: Supplementary file 1 [file Supplementary_file_1.docx]

Supplementary Material-1

**Supplementary Table 1 Search queries**

| **Database** | **Set** | **Search query** |
| --- | --- | --- |
| **Web of Science** | #1 | (TS=("health education" OR "health promotion" OR "health literacy" OR "patient education" OR "health communication")) |
|  | #2 | (TS=(Ophthalmolog* OR Ophthalmic OR "Visual system" OR Eye OR Eyes OR Vision OR "Refractive Errors" OR Myopia OR Hyperopia OR Hypermetropia OR Astigmatism OR "Anisometropia" OR Strabismus OR "Ocular Motility Disorders" OR Esotropia OR Exotropia OR Hypertropia OR Amblyopia OR "Lazy eye" OR "Eyelid Diseases" OR Blepharitis OR Trichiasis OR "Entropion" OR "Ectropion" OR "Ptosis" OR "Chalazion" OR "Hordeolum" OR "Lacrimal Apparatus Diseases" OR "Dacryocystitis" OR "Nasolacrimal Duct Obstruction" OR "Dry Eye Syndromes" OR "Conjunctival Diseases" OR Conjunctivitis OR "Vernal conjunctivitis" OR "Allergic conjunctivitis" OR "Bacterial conjunctivitis" OR "Viral conjunctivitis" OR "Corneal Diseases" OR Keratitis OR "Corneal abrasion" OR "Lens Diseases" OR Cataract OR "Congenital cataract" OR Glaucoma OR "Pediatric glaucoma" OR "Congenital glaucoma" OR "Retinal Diseases" OR "Retinopathy of Prematurity" OR "Retinoblastoma" OR "Visual Acuity" OR "Color Vision Defects" OR "Vision Disorders" OR "Low Vision" OR "Blindness" OR "Optic Nerve Diseases" OR "Optic Atrophy" OR "Nystagmus" OR "Congenital nystagmus" OR "Eye Abnormalities" OR "Coloboma" OR "Eye Injuries" OR "Ocular trauma")) |
|  | #3 | (TS=(Child* OR "Preschool child" OR "Preschool children" OR "Early childhood" OR "Young children" OR Toddler* OR Kindergarten OR "Nursery school" OR Pediatric OR Paediatric)) |
|  | #4 | #1 AND #2 AND #3 |
| **Pubmed** | #1 | ("Health Education"[Mesh] OR "Health Promotion"[Mesh] OR "Health Literacy"[Mesh] OR "Patient Education as Topic"[Mesh] OR"health education"[tiab] OR "health promotion"[tiab] OR "health literacy"[tiab] OR "patient education"[tiab] OR "health communication"[tiab]) |
|  | #2 | "Ophthalmology"[Mesh] OR "Eye Diseases"[Mesh] OR Ophthalmolog*[tiab] OR Ophthalmic[tiab] OR Eye[tiab] OR Eyes[tiab] OR Vision[tiab] OR ("Refractive Errors"[Mesh] OR Myopia[Mesh] OR Hyperopia[Mesh] OR Astigmatism[Mesh] OR "Refractive Errors"[tiab] OR Myopia[tiab] OR Hyperopia[tiab] OR Hypermetropia[tiab] OR Astigmatism[tiab] OR "Anisometropia"[tiab]) OR (Strabismus[Mesh] OR Amblyopia[Mesh] OR Strabismus[tiab] OR "Ocular Motility Disorders"[tiab] OR Esotropia[tiab] OR Exotropia[tiab] OR Hypertropia[tiab] OR Amblyopia[tiab] OR "Lazy eye"[tiab]) OR ("Eyelid Diseases"[Mesh] OR "Lacrimal Apparatus Diseases"[Mesh] OR Blepharitis[Mesh] OR Blepharitis[tiab] OR Trichiasis[tiab] OR "Entropion"[tiab] OR "Ectropion"[tiab] OR "Ptosis"[tiab] OR "Chalazion"[tiab] OR "Hordeolum"[tiab] OR "Dacryocystitis"[tiab] OR "Nasolacrimal Duct Obstruction"[tiab]) OR ("Dry Eye Syndromes"[Mesh] OR "Conjunctival Diseases"[Mesh] OR "Corneal Diseases"[Mesh] OR Conjunctivitis[Mesh] OR Keratitis[Mesh] OR "Dry Eye"[tiab] OR "Dry Eye Syndromes"[tiab] OR Conjunctivitis[tiab] OR "Vernal conjunctivitis"[tiab] OR "Allergic conjunctivitis"[tiab] OR "Bacterial conjunctivitis"[tiab] OR "Viral conjunctivitis"[tiab] OR Keratitis[tiab] OR "Corneal abrasion"[tiab]) OR ("Lens Diseases"[Mesh] OR Cataract[Mesh] OR Glaucoma[Mesh] OR Cataract[tiab] OR "Congenital cataract"[tiab] OR Glaucoma[tiab] OR "Pediatric glaucoma"[tiab] OR "Congenital glaucoma"[tiab]) OR  ("Retinal Diseases"[Mesh] OR "Retinopathy of Prematurity"[Mesh] OR "Retinoblastoma"[Mesh] OR "Retinopathy of Prematurity"[tiab] OR Retinoblastoma[tiab]) OR ("Vision Disorders"[Mesh] OR "Color Vision Defects"[Mesh] OR "Optic Nerve Diseases"[Mesh] OR Nystagmus[Mesh] OR "Visual Acuity"[tiab] OR "Color Vision Defects"[tiab] OR "Vision Disorders"[tiab] OR "Low Vision"[tiab] OR Blindness[tiab] OR "Optic Nerve Diseases"[tiab] OR "Optic Atrophy"[tiab] OR Nystagmus[tiab] OR "Congenital nystagmus"[tiab]) OR ("Eye Abnormalities"[Mesh] OR "Eye Injuries"[Mesh] OR "Eye Abnormalities"[tiab] OR Coloboma[tiab] OR "Eye Injuries"[tiab] OR "Ocular trauma"[tiab])) |
|  | #3 | ("Child"[Mesh] OR "Child, Preschool"[Mesh] OR "Infant"[Mesh] OR Child*[tiab] OR "Preschool child"[tiab] OR "Preschool children"[tiab] OR "Early childhood"[tiab] OR "Young children"[tiab] OR Toddler*[tiab] OR Kindergarten[tiab] OR "Nursery school"[tiab] OR Pediatric[tiab] OR Paediatric[tiab]) |
|  | #4 | #1 AND #2 AND #3 |
| **CNKI**  **And**  **WanFang** | #1 | 儿童 OR 小儿 |
|  | #2 | 眼病 OR 近视 OR 弱视 OR 斜视 OR 屈光不正 OR 视力不良 |
|  | #3 | 科普 OR 健康教育 OR 健康传播 OR 健康促进 OR 宣教 OR 患者教育 |
|  | #4 | #1 AND #2 AND #3 |

****Supplementary Table 2 Geographical Distribution of Survey Questionnaire****

| **Province** | **Count** | **Percentage** |
| --- | --- | --- |
| Beijing | 94 | 28.66% |
| Hebei | 63 | 19.21% |
| Shandong | 23 | 7.01% |
| Guangdong | 22 | 6.71% |
| Jiangsu | 13 | 3.96% |
| Sichuan | 13 | 3.96% |
| Shanxi | 10 | 3.05% |
| Zhejiang | 9 | 2.74% |
| Hubei | 9 | 2.74% |
| Henan | 8 | 2.44% |
| Shanghai | 7 | 2.13% |
| Guangxi | 6 | 1.83% |
| Anhui | 6 | 1.83% |
| Liaoning | 5 | 1.52% |
| Jiangxi | 5 | 1.52% |
| Neimenggu | 5 | 1.52% |
| Tianjin | 5 | 1.52% |
| Fujian | 4 | 1.22% |
| Chongqing | 4 | 1.22% |
| Xinjiang | 3 | 0.91% |
| Jilin | 3 | 0.91% |
| Hunan | 3 | 0.91% |
| Heilongjiang | 3 | 0.91% |
| Gansu | 2 | 0.61% |
| Ningxia | 1 | 0.30% |
| Shanxi | 1 | 0.30% |
| Guizhou | 1 | 0.30% |

# ****Supplementary Figure 1****


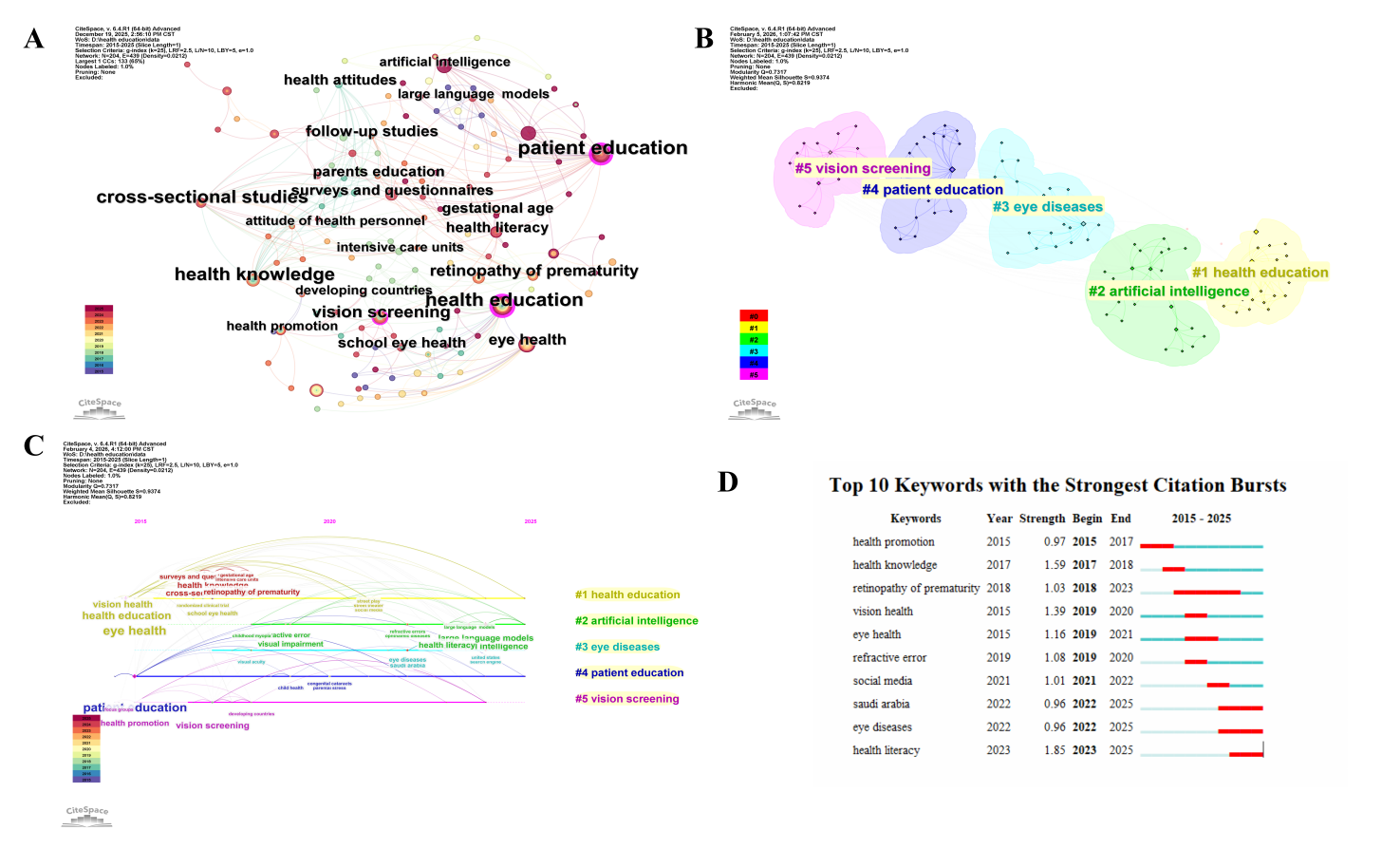


**Supplementary** **Figure 1:** Keywords analysis of Chinese database . (A) Keyword co-occurrenc visualization analysis on pediatric eye health education. (B) Keyword cluster analysis on pediatric eye health education. (C) The timeline view of keywords analysis on pediatric eye health education. (D) The top 10 Keywords with the strongest citation bursts.
