# Supplementary material for: Artificial intelligence driven transformation of pediatric eye health education based on bibliometric analysis and a cross-sectional survey
Source: Front Public Health. 2026 Mar 20;14:1781008. doi: 10.3389/fpubh.2026.1781008 (PMC13047839; doi:10.3389/fpubh.2026.1781008)
Supplement: Supplementary file 2 [file Supplementary_file_2.docx]

Supplementary Material-2

**Questionnaire Survey**

**Part 1: Demographic Information**

1.What is your child's age?

○ 1+ years old

○ 2+ years old

○ 3+ years old

○ 4+ years old

○ 5+ years old

○ 6+ years old

2. What is your child's gender?

*○ Female

○ Male

3. You are the child's:

○ Mother

○ Father

○ Grandmother (Maternal/Paternal)

○ Grandfather (Maternal/Paternal)

○ Other legal guardian

1. Your education level:

○ High school or below

○ Associate degree

○ Bachelor's degree

○ Master's degree or above

**Part 2: Profiles and perceptions regarding pediatric health education**

1.How frequently do you actively seek out information on pediatric medical knowledge?

○ Regularly and proactively

○ Occasionally, when encountered incidentally

○ Only when my child is ill

○ Rarely or never

1. Through which channels do you typically access information on pediatric medical knowledge? (Select all that apply)

□ Healthcare institutions (e.g., explanations from doctors/pharmacists, informational brochures/videos)

□ Online platforms (e.g., Baidu, Douyin, WeChat, Xiaohongshu)

□ Professional literature/books

□ Shared experiences from relatives and friends

□ Other (Please Specify): _________

1. What are the primary reasons for your limited familiarity with pediatric medical knowledge? (Select all that apply)

□ Lack of time to learn

□ Lack of interest

□ Limited exposure to such information

□ Perception of low utility/relevance

□ I consider myself sufficiently knowledgeable already

□ Other (Please Specify): _________

4.Have you ever explained basic medical concepts to your child?

○ Frequently

○ Occasionally

○ Almost Never

5.Do you believe it is necessary for children to learn basic medical knowledge? (Single choice)

○ Highly necessary

○ Moderately necessary

○ Unnecessary

○ Unsure

6.Which methods do you consider most appropriate for delivering medical knowledge to children? (Select all that apply)

□ Children's picture books

□ Animated videos

□ Expert-led talks/lectures

□ Interactive educational games

□ Other (Please Specify): _________

1. What aspects of current child-friendly medical science content are most in need of improvement? (Select all that apply)

□ A. Presentation is too dull, failing to engage children's interest

□ B. Content is not age-appropriate for my child (either too simple or too advanced)

□ C. Explanations are overly complex and difficult for children to comprehend

□ D. Content is not sufficiently engaging; children lack motivation to view/read it voluntarily

□ E. Concerns about content accuracy and potential misinformation

□ Other (Please Specify): _________

**Part 3: Guardian’s educational needs in pediatric eye health**

1. Which ocular conditions in children are you most concerned about? (Select all that apply)

□ Myopia (Nearsightedness)

□ Amblyopia (Lazy Eye)

□ Strabismus (Squint)

□ Astigmatism

□ Conjunctivitis

□ Trichiasis

□ Ocular Trauma

□ Other (Please Specify): _________

1. Please indicate your level of familiarity with the following pediatric healthcare topics:

Topic Unfamiliar Somewhat Familiar Very Familiar

Pharmacological/Methodological approaches for preventing childhood myopia? ○ ○ ○

Correct technique for administering ophthalmic eyedrops to a child? ○ ○ ○
